# Monthly Meeting of the College of Dentists, February 2

**Published:** 1858-07

**Authors:** 


### ARTICLE IX.

*Monthly Meeting of the College of Dentists, February* 2.

The President in the chair.

Mr. Felix Weiss read the following paper on the MANUFACTURE AND APPLICATION OF GUTTA PERCHA TO DENTISTRY:

Mr. President and Gentlemen.—In bringing before you a paper on "The Manufacture and Application of Gutta Percha to Dentistry," I fear I shall require all your kind indulgence; and indeed, were it not for the very liberal assistance I have received from many of the most experienced members of this College, I should have felt considerable timidity in introducing the subject at all. But in our at present comparatively imperfect knowledge of the many uses to which this valuable material may be applied, I consider that we cannot do better than solicit a statement of the experience and opinions of all those who have given it their attention, and the more so, as so much of our reputation depends upon the durability of the materials we are constantly called upon to recommend. Should the dentist introduce to the notice of his patient a material he considers applicable to their particular case, but at the same time with an honest statement of its imperfections, and that material prove, by experience, more perfect than was anticipated, he gains for himself credit, and establishes a confidence it must be our highest ambition to merit; but should he, on the other hand, highly extol that of which he has no experience, resting his convictions simply upon the assurance of one, perhaps, as inexperienced as himself, the danger to his reputation is very great indeed, and he had better have continued in the beaten track, with honor to himself and safety to his patients; but, thanks to the spirit of emulation that is among us—thanks to the formation of this College and the opportunity it gives us freely to lend our experience and impart all those little trifles our daily practice brings before us—I say, thanks to these advantages, we can no longer collectively be deceived; and if we are, *individually*, it is because we have rested too much upon our own knowledge, instead of submitting our views to the calm consideration of the many.

These remarks apply particularly to the subject of my paper, for this material, gutta percha, I must freely admit offers many temptations to the young, the hasty workman, and the charlatan. Gutta percha may be likened to putty in the hands of an unskillful carpenter. Should a side block be inaccurately let down, line it with gutta percha: should a plate be carelessly fitted, line it with gutta percha. Teeth may be raised on it, and indeed, it may be applied to disguise every imperfection, and yet the piece of work be worn by the patient with comparative comfort. But let me here remark what is known to all—nothing can compensate for inaccurate fitting; nothing will excuse carelessness of construction, and nothing, in the eyes of an honest practitioner, atone for a want of scientific arrangement, and workmanlike finish.

I shall divide my subject into four heads:

1. The manufacture of gutta percha.

2. Its application to dental mechanism.

3. Its great use in temporary pieces, and in cases of emergency.

4. General remarks upon its durability, &c.

Gutta percha is imported into this country in the form of large blocks, of more or less purity. Some are so incorporated with dirt, coloring matter and woody fibre, as to be unfitted for the dentist's use, unless previously submitted to a chemical operation.

Its mode of preparation is either by solution and after-evaporation, or by boiling in water and mechanically removing its impurities.

In preparing it by solution, all that is necessary is to confine small portions in a piece of fine linen, and place the whole in a bottle of chloroform or other fluid which will readily dissolve it; pour off the clear solution and evaporate. It is placed in linen to free it from pieces of bark, wood and other impurities, which are so large as to be separated by filtering, and with which it is generally found mixed. I prefer, however, to take the gutta percha which has been previously boiled and cleaned. I shall not dwell longer on this mode of preparation, for unless we gain a greater amount of durability, I cannot see upon what principle we should advocate a process, not only expensive, but also exceedingly tedious: to this point, I shall, however, again return at the end of my paper. The second method of preparing gutta purcha is as follows:—select a block, which, upon being cut through, presents a white surface; and I may here remark, that were the consumption in any way considerable, I feel convinced it might be collected with greater care, and save a vast amount of time and labor.

Take a piece weighing about two or three ounces, peel off with a sharp knife the outer rind; do so freely until you have removed the light brown covering. I prefer doing this before boiling; place it in an enameled pan or glass vessel, and boil it freely; it is much more convenient to do this over a gas lamp constructed for the purpose: after it has been boiling about five minutes, take it out and examine the outer side to see that you have cut away all the rind, and commence kneading it into a sheet. If the gutta percha is a fine specimen, it will assume a creamy white appearance, free from specks, and after boiling once or twice, and kneading, may be mixed with a small portion of carmine. Some object to the use of this coloring agent, as it is said not to keep its color so well as the purest forms of rose pink.

But it frequently happens that a block cannot be obtained so pure, and it will then be necessary to draw it out until it is as thin as paper; and by holding it up to the light, every little speck may be detected, and must be carefully cut out, and when thoroughly washed and kneaded, may possibly still remain one shade darker than the preparation first mentioned. It is hardly necessary to add, that in all operations with gutta percha, the greatest attention must be paid to cleanliness; every vessel used should be perfectly clean, and the gutta percha not handled more than necessary. One peculiar property is the darkening influence of air, light and heat. After the fourth or fifth washing, every application of heat has a tendency to darken it, and a piece, which presented an excellent appearance when first prepared, may, after exposure to the light and air for some weeks, get many shades darker and will assume a motley appearance; this some endeavor to prevent, by keeping it in water and in the dark, although by doing so its color is neither improved, nor its disagreeable smell in any way corrected. After being carefully prepared, I have found it better simply to enclose it in two or three folds of paper, although it is advisable not to make it up in too large a quantity at a time. Generally, the whitest specimens are the most disagreeable in smell; indeed it is very rarely that the brown gutta percha has any smell at all; but this can be removed by washing and free exposure to the air in thin sheets, for a few days.

The method of applying gutta percha to mechanical work is so generally understood, that I shall not detain you very long in describing it. Having taken your model and run it in plaster, the first thing is to cast one in zinc. You then take a piece of sheet wax, of the thickness you wish your gutta percha to be when finished, (that used by wax flower makers will do very well for the purpose,) generally about the thickness of half-a-crown will be sufficient, but this must depend upon the case. You place the wax upon your plaster model and cut it to the size you wish your plate to be, then bevel the edges, so that the line of the plate on the gum may be only the thickness of the gold. This model you cast in zinc also, and make your plate to, so that when the wax is taken away you have a space underneath to receive the lining of gutta percha. Into this space you spread a small strip of platina wire gauze, which must be soldered round the edges; you then mount your teeth and blocks in the ordinary way. When the case is completed, and the teeth all fastened on, you take a sheet of gutta percha, and having well softened it in hot water, press it into the plate ; then wet the zinc model first taken, and with considerable force press the set well down into its position—you may have occasion to soften the gutta percha in water many times, to do this effectually; afterwards, with a knife warmed in the flame of a spirit lamp or a gas lamp, burning a mixture of air and gas, trim off all that is superfluous. It is necessary to cut clean, and do not attempt to drag the gutta percha. Considerable nicety is required in the trimming. Sometimes the gutta percha, and particularly in lower sets, is brought round the fronts of the teeth, and nicely trimmed round them ; the work, before being placed in the mouth, requires to be warmed in hot water, which allows the gum to take the last fine fitting ; and when all is completed to your satisfaction, polish by gently rubbing with the thumb all those parts which have been cut with the warm knife. If this is nicely managed, with a little practice, a beautiful polish will result. It is necessary to see that no straggling ends remain, but all should be finished off to the greatest nicety. Now all cases, upper or lower, are modifications of this arrangement. Sometimes, in lower sets, the gutta percha is brought round the backs of the teeth, as well as the front, so that no plate at all is seen ; but generally, both in upper and lower cases, it is well to let the plate be exposed at the back of the teeth, as the gutta percha is more liable at this part to be abraded.

Modifications of this arrangement will be readily devised by any practical dentist; for instance, some might prefer, instead of the wire gauze, using a lining of thin plate, perforated with a number of holes—at the same time, as it offers a hold for the gutta percha, serves to strengthen the plate.

We may require to use the gutta percha for bands alone; this may be done by turning a thin piece of plate into the form of the letter C and with this making a band, so that the open side may receive the gutta percha, or a piece of oval tubing may be used instead, and by afterwards cutting a portion from the inner side, an opening will be made, which will securely hold the lining of gutta percha.

Gutta percha will be found, in some cases, of the greatest use in pivoting stumps which have become mere shells. Where the hole is very large, the pin should have a small strip of fine wire gauze soldered round it, and the hardest description of gutta percha well pressed into its meshes; if the cavity be smaller, the pin should be split, by sawing it down the centre, and fining off the ends and soldering them. After being accurately fitted and the superfluous gutta percha cut away, the stump should be wiped out, and painted with a solution of gutta percha in chloroform, the plug slightly warmed, *without using water*, and driven into its place. I have seen a pivot thus fixed remain perfectly secure for a twelvemonth, after all other modes of pivoting had become useless.

Although my experience does not warrant me in advocating the application of gutta percha to bone for permanent sets, yet it is so invaluable for temporay purposes, this paper would be hardly complete without some allusion to it. Holes should be drilled about one-eighth of an inch in depth, in that part of the bone intended to be lined; some caution must be exercised in drilling only into the thickest portions of the bone, underneath the blocks, &c.; each of these holes should be slightly excavated, so as to form a cavity, into which the gutta percha may be driven, but from which it would be difficult to draw it. These cavities should then be separately stopped with warm gutta percha, and the bone kept perfectly dry. A sheet may then be softened at the fire or over a lamp, and apply to the bone, which has been previously warmed; by this means the gutta percha may be made to hold to the bone with considerable firmness. My reasons for not advocating its general adaptation will be given hereafter.

I have now, I think, very briefly alluded to the different classes of dental mechanism, to which gutta percha is more generally applied, and unquestionably the preference must be given to that composed of gold and gutta percha, more particularly in lower sets; but it is in temporary pieces, and in cases of emergency, that its great value will be found. While the gum is absorbing and the mouth changing, it furnishes a material which can be modeled and remodeled at very little cost or labor, and secures for our patient a well fitted piece of work throughout the long and tedious interval between removing teeth and fixing in artificial ones permanently.

I thall now take the liberty of drawing your attention to four classes of patients for whom gutta percha offers advantages they could not in any other material with which I am acquainted obtain.

The first is the case of a gentleman who suddenly breaks an upper bone set; he has to attend to an appointment of the greatest importance in two hours time; he has to speak and he cannot do so without his teeth. In the greatest distress he begs that you will endeavor to do something for him. The bone is so softened, no rivets will stand, and it is a hopeless case. Having taken a model, see whether among all the bone pieces you possess (and we most of us have a number of carved sets, the practice of our younger days) you can find any thing like the upper he has fractured. It is of little moment whether the teeth are carved or set on, so that it is perfect; having found such a piece, rough it to the model, drill your holes and fix in the gutta percha lining, warm and fit it to the mouth, carefully cut down your bite, and your case is completed. In one hour and a half your patient may be restored to comparative comfort; he can at least speak, and keep his appointment; you have plenty of time given you to make a new set. I succeeded so well in a case of this description, that my patient declared he had not suffered one moment's inconvenience.

Second. A person wearing a large upper gold plate, with several teeth, loses the supporting tooth on the one side; the plate will not hold up, and they are consequently seriously inconvenienced. Drill a number of holes, about the eighth of an inch from the edge of the plate, sink them a little in the face, also drill a few holes between the teeth; with a pair of pliers bend the edge of the plate slightly towards the gum, so that it may not feel too thick at the edge, and proceed to press in a lining of gutta percha, trim with a warm knife, and see that the gutta percha has passed through the holes, which secure with a hot iron. The gutta percha having been softened, the plate must be carefully placed in the mouth, and may require a little trimming afterwards.

Third. We have all found cases in which the mouth has become so contracted, that taking models has not only been a very troublesome, but a very painful task. In one case the lady had found such difficulty, even in removing the set from the mouth, that she let it remain there for months together, and the mouth had eventually become so contracted it caused her the greatest agony in attempting to take a model, the jaw being unusually large. In this case, models were taken from the set in wear—a gold one, and a gutta percha set made, which, as it took its own finishing impression, answered admirably.

Fourth. A gentleman suffering from chronic bronchitis, the moment the wax was put in the mouth, was attacked with the most alarming coughing and reaching, compelling you to desist; and a set had to be constructed in gutta percha from models taken ten years ago, yet when completed was perfectly successful.

These, gentlemen, are a few of those troublesome cases we sometimes meet with, and I cannot too warmly extol the advantages possessed by gutta percha, in overcoming difficulties of this character, its ready adaptation, the simplicity with which any degree of elevation can be obtained, its lightness and accuracy of fit, cannot be too much praised; would that we could also add its durability; and this brings me to that head, which I must consider the most important of my subject, and it is here, gentlemen, I beg of you to assist me—to give to the profession the experience of each individual practitioner, and this I conceive to be one of the great advantages we possess at the present day; meeting together, our aim should be, the diffusion of all those little minor facts, which united, approach perfection. That the subject of my paper deserves your deepest attention, is exemplified in the different opinions entertained by different practitioners. A member of your College, holding an excellent position, with every opportunity of testing the properties of the material I am calling your attention to, writes, that in every instance he has used gutta percha, he has been greatly disappointed, and dwells strongly on its many disagreeable qualities. Another equally well informed member assures me, that in no case has gutta percha been otherwise than perfectly successful, and he feels quite justified in recommending it for a certain class of cases. I feel convinced, that the time has arrived, when the question—Is gutta percha to be recommended?—can it be employed in the mouth with benefit to the patient, and credit to the practitioner? should receive an answer. Gentlemen, I unhesitatingly declare, that could gutta percha be prepared, which would stand the test of a number of years in the mouth without change or unpleasantness, it would surpass every other material where its bulk is not against it. How immeasurably superior to bone—how much easier to manage!

In carefully sifting the evidence I have been able to obtain, united to my own experience, I have come to the conclusion that in its present form, gutta percha cannot be recommended as lasting more than from two years to two years and six months, in the generality of mouths. I consider it nesessary that it should be renewed every two years, or three at the outside; but in this remark I am alluding to the soft white gutta percha prepared simply by washing; and here again we are in doubt, some considering the soft as more durable than the hard. Mr. Jacobs, of Bridgewater, has most kindly forwarded to me several specimens, prepared by solution in chloroform, and he has particularly observed, that the white specimens of gutta percha are much the softest, and as he believes the less durable. He is inclined to think that the gutta percha purified by solution in chloroform would be more durable in the mouth than the raw material, as containing no extraneous matter, and being solid, homogeneous, and free from all porosity. Now does the fact of its being purified by chloroform free it from all porosity? I should say not; it may lessen it, but its general character must remain unchanged. Under the microscope I cannot discover any structural change; both that prepared by washing, and that by solution in chloroform, present the same dense close texture. It appears to me, that it is its porosity constitutes its greatest drawback. From the experiments I have made, and the opinions conveyed to me by some of our most experienced practitioners, I am led to believe, the liquids of the mouth and finely divided particles of food, become so impregnated in the gutta percha, as to cause the very disagreeable smell it sometimes acquires.

I have my doubts, whether gutta percha is decomposed in the mouth. The lining of a plate which had become very foul, and the surface slightly abraded, having been worn a little over two years, was boiled and kneaded up several times; it lost its disagreeable smell entirely, and excepting in being some shades darker, appeared very similar to when placed in the mouth. I have seen a plate lined with gutta percha, that had been worn constantly for two years, and yet was not in the slightest degree tainted; but we know how differently bone is affected in different mouths, and gutta percha must be similarly tested. Bone, in contact with gutta percha, decomposes most rapidly, and the reason of this can be easily understood; when the bone becomes thoroughly moistened, it has a tendency to recede from the gutta percha, it becomes slightly expanded, finely divided particles of food get between, and decomposition sets in rapidly.

I cannot advocate the use of gutta percha bands; no matter how constructed, they appear to have a tendency to cause sensitiveness of the necks of the teeth, and accumulating, as they must do, particles of food, cause them to decay much more rapidly. In conclusion, gentlemen, if I might be permitted to suggest what I should consider the most durable, and the best suited for applying to dental mechanism, it would be the finest gutta percha, thoroughly purified, and mixed with silica, or some other ingredient, as recommended by Mr. Jacobs. He informs me, that as a stopping, he has used it from six to seven years, he has seen many cases done three or four years ago, and which still remain quite perfect. If this form of gutta percha will stand in a tooth, why not applied to mechanism? As a stopping for children's first teeth, gutta percha, somewhat similarly prepared, I have repeatedly used with the very best results. Let us then unite the experience we have each of us collected, add item to item, and believe me, the period is not far distant, when gutta percha may be placed in its true position, either added to our stock of every day materials, or cast aside as useless. In thanking you, Mr. President and gentlemen, for the kind attention you have given me, I cannot conclude, without at the same time, thanking those members of your College to whom I have referred, and by whom I have been received with the greatest cordiality, more particularly, I would thank Mr. Faulkner. The time was, when we may have had secrets among us, or at least we fancied we had; it should now be our proudest endeavor to dissipate all such antiquated notions, and by the readiness with which we lend our experience, raise our profession to its true position.

I must apologize for the cursory manner in which I have been compelled to run over the subject. I cannot claim for myself praise, as having introduced any novelty, or enforced any new application, but I feel every satisfaction in having introduced a subject, worthy, I am convinced, of the attention of every member of our profession.

*Discussion.*

The President said, that gutta percha, for dental purposes, was introduced in America about ten years ago, and he believed it had received a very general trial by practitioners there. He had used gutta percha combined with silica, as a temporary stopping for children's teeth, and for such purposes he had not the slightest hesitation in pronouncing it invaluable.

Mr. Rymer inquired, whether Mr. Weiss made use of gutta percha as adapted to artificial teeth generally, or only in specific cases?

Mr. Weiss.—Not for general purposes.

Mr. Fox thanked Mr. Weiss for the manner in which he had brought forward his paper, and remarked that the meeting was as much indebted for the method the essayist had adopted, as for the interesting subject matter itself.

Mr. W. G. Bennett said, in using gutta percha, he had observed two varieties; one would work with a smooth surface, the other always appeared somewhat rough and rotten. Could Mr. Weiss inform him of any ready means of converting the rough variety into the smooth?

Mr. B. also observed, that he remembered three cases in particular, in which gutta percha had been applied to bone, and where it appeared to exercise a preservative rather than a destructive influence. The first case was a bone lower piece for two incisors, and the molars on each side; this piece was filled with gutta percha at the part in contact with the necks of the remaining teeth, and also the gum under the incisors and molars. About a dozen holes, the size of the tubes of mineral teeth, were drilled in the bone, and countersunk. A mould was made of plaster of paris, hardened with white wax, and fixed into two iron frames, connected by guide-pins, and acting in the manner of ordinary casting frames. The mould, with the bone piece, and softened gutta percha in it, was then submitted to the action of a powerful screw-press. When this piece had been in the mouth for at least six months, the gutta percha adhered closely to the bone, and there was no sign of decay at the parts where the bone and gutta percha were united.

The second case was an entire upper suction piece ; eight teeth in front were mounted on platina, with continuous gum, a solution of gutta percha in chloroform was applied between the bone and the platina. In this case the bone was much better preserved than when a solution of mastic, in spirit, was used.

In case the third, a like solution of gutta percha was placed under the side blocks of a gold upper piece, which had been repaired ; when it was observed some months aftewards, the blocks were quite sound, except at about a quarter of an inch of the edge of one of them, where the gutta percha appears to have been accidentally omitted.

Mr. Rogers said, that he always used gutta percha, and he did so from the perfect success he had been fortunate enough to obtain ; with regard to its durability, he had been able to test that, and could mention a case where it lasted six years, which he considered ample time. The failures which had attended its use by many practitioners, arose, no doubt, from the impurity of the material employed, for he knew that the depots did not furnish gutta percha so pure as it might be. He had not found the inconvenience alluded to by some arising from the offensive smell they described, and this he also attributed to the genuineness and purity of the gutta percha. He might stand alone in his opinion ; but it was his opinion, nevertheless, that dentists did not succeed in fitting the mouth—they caused the mouth to fit the plate, but he maintained, that they did not positively and actually fit the mouth ; but where gutta percha as a lining to the plate was employed, this very desirable end was very readily attained.

Mr. Harley said, he did not like gutta percha at all ; he had always been able to work both gold and bone to the advantage of his patients, and he considered this new material altogether unnecessary. It might do very well to patch up slovenly work, and rectify misfits ; and he believed that, in such cases, bad workmen would use it.

Mr. Purland objected entirely to Mr. Rogers' remarks respecting the fitting of the mouth—he had always been able to fit his patients' mouths ; in fact, he rather prided himself upon his ability to do so, and he should very much question, whether a model could be produced which he could not fit. With regard to Mr. Harley's remarks, he begged to say, that he invariably took two models, one of which he retained in his own keeping, and by which he tested the fit after the piece had left the work-room, which entirely prevented his being deceived.

Mr. Hill said, that like all other novelties in the profession which he had been able to obtain, he had endeavored to give gutta percha a fair trial, but he could not say that he was very much satisfied with the results. The material he employed had been purchased at the depots where he had hoped to obtain the best; whether it might be impure, as Mr. Rogers had stated, he could not say ; but he had found it highly offensive after it had been in the mouth a very short time. In one instance, he noticed a remarkable coating of tartar upon the lower frame-work of a piece lined with gutta percha, which did not appear in the companion piece made of gold and mineral teeth ; and from this he gathered, that the porosity of gutta percha was its chief drawback, allowing, as it did, the secretions of the mouth, and very fine particles of food, to find a lodgment about its surface. He only used it in temporary cases.

Mr. Rymer said, he had not had much experience in the use of gutta percha, because he had found the use of gold and bone to answer so well almost all the purposes of mechanical dentistry, that he considered these materials could scarcely be superseded. In some few instances, he had employed gutta percha for temporary purposes, but beyond this, he should not feel inclined to use it. In reference to the case alluded to by Mr. Weiss, of a person who had worn his old set of teeth so long that the lower broke just at a time when the wearer required an immediate repair, and that repair was rendered impracticable from the softness of the bone, he could only say that the patient almost deserved to be put to inconvenience, for having delayed so long having a new set.

Mr. Hockley said, he had used gutta percha with very variable results, but he was compelled to admit, that when it had answered best, it was Mr. Truman's material that he had employed.

After some observations from Mr. Ghrimes in favor of the use of gutta percha—

Mr. Weiss, in reply, accounted for the diversity of opinions expressed by those gentlemen who had spoken, as to the durability of gutta percha, from the fact of there being so many varieties; and, in answer to Mr. Bennett, remarked, that a very good method of testing its toughness was by biting off a small piece, which could be flattened out, even without heat, if the specimens were worth anything. He could not go so far as Mr. Rogers, and recommend its being employed in every case; he considered it particularly applicable for complete lowers; indeed, he would never think of employing any other material when there had been much absorption. He must differ from Mr. Harley; his remarks might be applied to every new material introduced, and put a stop to the spirit of advancement altogether. Gutta percha would be found, by those who applied it, something more than a patch upon slovenly work, and he felt convinced, both Mr. Hill and Mr. Hockley would have arrived at more generally favorable conclusions, had they prepared their own material. He wished distinctly to state, that when the gutta percha was pure, even though it did decompose in time, it was not attended with more unpleasantness than in the case of bone; and he had not yet found one instance where the patient complained that the stomach was at all affected by that decomposition. Still, the opinion he had expressed in his paper had not been changed, and he should advise that the gutta percha be renewed every two years, if practicable.

A cordial vote of thanks was passed to Mr. Weiss for his able paper.

Some specimens of "Cast Plates" were exhibited by Mr. J. L. Statham, which were briefly commented upon by Mr. Fox; after which the meeting was adjourned to Tuesday, March 2d.—*Lon. Quar. Jour. Den. Sci.*

